# Nurse Educators' Background, Education, and Experience in Digital Competence Profiles: A Descriptive Comparative Cross‐Sectional Study in Four Countries

**DOI:** 10.1111/jan.70077

**Published:** 2025-07-15

**Authors:** Juha Pajari, Marjorita Sormunen, Leena Salminen, Imane Elonen, Miko Pasanen, Leandra Martin‐Delgado, Michelle Camilleri, Andrea Sollárová, Elaine Haycock‐Stuart, Terhi Saaranen

**Affiliations:** ^1^ Department of Nursing Science Faculty of Health Sciences, University of Eastern Finland Kuopio Finland; ^2^ Institute of Public Health and Clinical Nutrition Faculty of Health Sciences, University of Eastern Finland Kuopio Finland; ^3^ Department of Nursing Science University of Turku Turku Finland; ^4^ Turku University Hospital Turku Finland; ^5^ Turku University of Applied Sciences Turku Finland; ^6^ Department of Nursing Faculty of Medicine and Health Science, Universitat Internacional De Catalunya Barcelona Spain; ^7^ Department of Nursing Faculty of Health Sciences, University of Malta Malta; ^8^ Department of Nursing Constantine the Philosopher University in Nitra Nitra Slovakia; ^9^ Nursing Studies School of Health in Social Science, University of Edinburgh Edinburgh UK; ^10^ Elsie Inglis Quad, The Medical School Edinburgh UK

**Keywords:** cluster analysis, competence, digital technology, education, nursing

## Abstract

**Aim:**

To identify and compare the digital competence profiles of nurse educators, the background variables associated with profiles, and the self‐assessed level of digital competence in four European countries.

**Design:**

A descriptive comparative cross‐sectional study.

**Methods:**

Data were collected from nurse educators (*n* = 263) in 36 nursing education organisations in Finland, Malta, Slovakia and Spain. Partitioning around medoids (PAM) clustering was used to identify competence groups, and descriptive and inferential statistics were used to examine the association of nurse educators' background variables.

**Results:**

The clustering analysis resulted in two nurse educator digital competence profile groups: high and moderate. The profiles differed based on completed pedagogical studies and teaching experience, with an emphasis on the high competence profile. Educators in the high competence profile group showed greater interest in using educational technology and self assessed their digital competence at a higher level compared to educators in the moderate competence profile group. Nurse educators' lowest digital competence was in the safe and responsible use of technology, such as knowing copyright laws.

**Conclusion:**

Despite the heterogeneous background of nurse educators, international continuing professional development needs in digital competence are identified. Nurse educators' continuing education should support the utilisation of technology through pedagogical approaches, and educators' competence in the safe and responsible use of technology (e.g., how to protect digital materials) must be enhanced in nursing education organisations.

**Implications for the Profession:**

This study highlights the need to further develop nurse educators' digital competence. Continuing professional development should target preparation in safe and responsible technology use and include pedagogical studies and mentoring from experienced peers.

**Reporting Method:**

The STROBE checklist was adhered to in reporting the results.

**Patient or Public Contribution:**

Each participating educational organisation assigned a contact person to distribute the survey to the nurse educators.

**Summary**

- What problem did the study address?
  ○ There is a lack of common continuing professional development opportunities to enhance nurse educators' digital competence. This was the first study to examine nurse educators' digital competence profiles across four EU countries in order to target educators' continuing education.
- What were the main findings?
  ○ From the self‐assessed digital competence responses of nurse educators from four European countries, cluster analysis identified high and moderate digital competence profiles. Educators in the high digital competence profile had mostly completed pedagogical studies and had more teaching experience. They were more interested in educational technology and rated their own digital competence level higher than that of educators in the moderate competence profile. A key area to be addressed for nurse educators is the development of their competence for the safe and responsible use of technology for themselves and their students.
- Where and on whom will the research have an impact?
  ○ The results of this study can be utilised internationally in nursing education organisations. When planning nurse educators' continuing professional development activities, it is important to include technology‐integrating pedagogical approaches in continuing education. Peers with higher digital competence and an interest in educational technology could be utilised for developing mentoring programmes with colleagues. The content of continuing education for nurse educators should focus on the safe and responsible use of technology, such as supporting students to protect sensitive digital material.
- What does this paper contribute to the wider global clinical community?
  ○ To implement modern nursing education, nurse educators require digital competence to utilise evidence‐based technological teaching, learning, and assessment methods. Educators' continuing professional development activities, such as continuing education, should meet these digital competence needs. Despite the heterogeneous background of nurse educators by country, this study's results identified two digital competence profiles that can be used internationally in the targeting of continuing education for nurse educators.
  ○ The completed pedagogical studies and acquired teaching experience of the nurse educators may explain their higher level of digital competence. In educators' continuing education, the study of pedagogical approaches could be justified, thus enabling versatile insights into the integration of technology into today's nursing education. Educators with higher digital competence and interested in educational technology could participate in the implementation of continuing education for their peers through mentoring, for example.
  ○ Nurse educators' digital competence could be enhanced as part of continuing professional development, especially in respect of the safe and responsible use of technology. This competence is important with regard to modern nursing education and practice, as various sensitive materials are produced and used, such as videos and registry data. As technology continues to evolve, nursing education organisations should ensure that nurse educators have the competence to teach students these safe and responsible digital practices.

## 1 Introduction

The digital competence of educators has become essential in modern educational environments (The Organization for Economic Co‐operation and Development [OECD 2023]). This competence to utilise technology in teaching, learning, and assessment is necessary because healthcare education and practice demand professionals to be able to manage developing working methods (Filej et al. 2024). These development needs for nurse educators have become more evident—for example, with the expansion of hybrid education, including the implementation of varying digital teaching methods, learning materials, and solutions to support students' safe interaction (Jämsä et al. 2024; OECD 2023). Continuing education for nurse educators should correspond to these evolving digital competence needs.

Continuing professional development (CPD) is a self‐directed learning process in which professionals reflect, identify, and develop their competence by engaging in various learning opportunities, such as continuing education (Drude et al. 2019). The opportunity for CPD has been seen as a possibility to respond to the career advancement and mobility of healthcare professionals (Directive 2013/55/EU 2013; World Health Organization [WHO] 2021) and as one way to solve the shortage of nurse educators (Gazza 2019; Vardaman et al. 2024). The challenge in organising and participating in continuing education is limited resources (Brewer et al. 2024). Therefore, there may be a reason to prioritise education based on competence needs (Brewer et al. 2024; Pilcher 2016). Because nurse educators' educational structures and competence vary by country (Campos Silva et al. 2022; Elonen et al. 2023), it is necessary to identify groups based on the digital competence level. With this knowledge, it is possible to target continuing education in response to learning needs to enhance nurse educators' digital competence (Drude et al. 2019; Jobst et al. 2022; Pilcher 2016).

## 2 Background

The implementation of targeted and appropriate continuing education for CPD may require an understanding of nurse educators' heterogeneous background. The reason for this heterogeneity can be accredited to the absence of uniform legal regulations for nurse educator education (Satoh et al. 2020; Leighton et al. 2022; Wells‐Beede et al. 2023). As Campos Silva et al. (2022) stated, these qualification requirements, standards and educational pathways vary across countries (Campos Silva et al. 2022). Even the definition of a nurse educator varies universally (Zlatanovic et al. 2017; Satoh et al. 2020). When defining a nurse educator, it should be noted that there are other health‐related teaching professionals, such as clinical nurse educators and health education teachers (Kaarlela et al. 2022; Paakkari et al. 2024). With these different professionals, the context of the teaching differs (e.g., regarding the learning needs of the group being taught), leading to a variation in the qualification requirements (Leighton et al. 2022; Paakkari et al. 2024).

In addition to the lack of uniform legal regulations and varying definitions of a nurse educator, several competence descriptions exist for nurse educators (Campos Silva et al. 2022). One of these is the WHO's *Nurse Educator Core Competencies* (WHO 2016). More local country‐specific ones also exist, such as *NLN Core Competencies* for nurse educators in the US, published by the National League of Nursing (Fitzgerald et al. 2020; NLN 2022) or the health and social care educator's competence model in Finland (Koskimäki et al. 2022; Mikkonen et al. 2021). However, educational organisations lack common practices for integrating the contents of these competence descriptions into the curricula for nurse educators' education. This inconsistency can contribute to varying levels of competence together with the heterogeneous background of nurse educators (Campos Silva et al. 2022; Fitzgerald et al. 2020).

Due to this heterogeneity of nurse educators' backgrounds, it may not be possible to create a global and uniform continuing education programme for a nurse educator. Nevertheless, discussion of the harmonisation of education and, thus, competence is considered a common agenda (Gazza 2019; Satoh et al. 2020; WHO 2021). For example, international cooperation to plan and implement healthcare education, such as remote joint teaching or staff and student movement, creates a need for a common understanding of nurse educators' digital competence levels (WHO 2021; Directive 2013/55/EU 2013; Filej et al. 2024).

Existing nurse educators' competency descriptions, such as the previously mentioned WHO guidelines, view technology as part of the educator's work, but a more detailed description of digital competence is missing (Krumsvik 2022; Tischendorf et al. 2024). For example, the use of technology to support the teaching and learning process (WHO 2016) or the ability to design versatile virtual learning environments and manage digital technology for collaboration (Mikkonen et al. 2021) has been noted. However, in the context of education, a wide variety of comprehensive frameworks about digital competence exist (UNESCO‐UNEVOC 2024). These various existing frameworks and the concept of digital competence are affected by the rapidly evolving development of technology. For this reason, there is a necessity to define digital competence for the contexts in which it will be used (Krumsvik 2022).

In this study, digital competence is understood in accordance with the European Framework for the Digital Competence of Educators (DigCompEdu), where an educator's digital competence consists of six competence areas, including 22 competence descriptions (Redecker 2017). This framework, published by the European Commission, has been widely applied to varying educational contexts from early childhood to higher and adult education (Santos et al. 2022; Zhao et al. 2021). Applying the framework to this study, digital competence refers to how nurse educators integrate technology to enhance their professional practice; empower teaching, learning, and assessment; and improve learning outcomes as part of their work in nursing educational organisations (Redecker 2017).

Additionally, this integration of technology is seen to be more than updating accustomed ways of working. For example, meeting a student can now take place remotely (de Gagne 2022; Jämsä et al. 2024). Effective utilisation of evolving technology, such as artificial intelligence for assessment (Xia et al. 2024) and online environments for interaction (Männistö et al. 2020), requires a complete rethinking of the pedagogical approaches (de Gagne 2022). Among other things, the responsible use of artificial intelligence in the preparation of materials and assignments must be recognised (Xia et al. 2024). Despite the rapid development of technology‐based approaches, these have been shown to be applicable for nursing education (Breytenbach et al. 2017; Männistö et al. 2020; Sormunen et al. 2022). However, educators must first adapt to the use of this technology (Filej et al. 2024), which demands consideration of the resource barriers, such as a lack of scheduled time for continuing education (Brewer et al. 2024). Moreover, the educators retain responsibility for safe, reliable, and evidence‐based teaching, learning, and assessment methods. Simultaneously, they need to consider students' learning needs and ownership of technology, such as the usability and accessibility of digital materials on available devices (Breytenbach et al. 2017; Jobst et al. 2022; Oermann 2022).

Overall, educators' digital competence has been narrowly studied in higher education (Santos et al. 2022; Zhao et al. 2021), and nurse educators' level of digital competence has been examined in only a few publications (Boté‐Vericad et al. 2023; Lee and Bello 2023; Li et al. 2025; Pajari et al. 2022, 2024; Ryhtä et al. 2021). Based on these studies, the COVID‐19 pandemic required the adoption of new technological work methods, and nurse educators' digital competence appears to be of an average level. The most advanced expertise has been in enabling collaboration by utilising technology, and educators have shown interest in developing their digital competence. Some studies have identified development needs, such as familiarisation with copyright laws when creating and sharing digital learning materials (Boté‐Vericad et al. 2023; Pajari et al. 2022). In addition, nurse educators were seen to require more versatile competence when supporting students' integration into the technology‐rich clinical nursing environments (Li et al. 2025).

Similarly, the association among the several background variables of nurse educators in respect of their digital competence has been scarcely researched. The experience of teaching online (Lee and Bello 2023) and the age of educators have been found to be partly related to digital competence (Li et al. 2025; Pajari et al. 2022). In the context of healthcare professionals, in addition to age, graduation year, and attending orientations for technology use at work (Kaihlanen et al. 2023), the overall interest in using technology (Jarva et al. 2024) has been associated with the competence to utilise technology. However, a more comprehensive comparison of digital competence between groups must be undertaken to understand nurse educators' CPD needs.

It should also be noted that some views of digital competence have been included in literature reviews (Smith et al. 2023; Tischendorf et al. 2024) describing the continuing education of nurse educators or healthcare professionals. Those results showed there is limited, if any, identifiable continuing education for healthcare education professionals to develop their digital competence. Smith et al. (2023) revealed that nurse educators' continuing education is fragmented without structure. The utilisation of technology was noted in communication, collaboration, and professional competencies. In the article included in the review (Koskimäki et al. 2022), nurse educators highlighted the lack of existing continuing education that meets their needs and the necessity for pioneering continuing education.

Otherwise, the digital competence in healthcare education has rarely been the subject of CPD and continuing education research. Vähäkangas et al. (2022) revealed that the utilisation of technology in pedagogy is one of the key needs of educators' continuing education. Moreover, studies have usually focused, for example, on evaluating the implementation and impact of continuing education (Marceau et al. 2024). The results of these studies can be used when planning, implementing, and evaluating continuing education for nurse educators, which can be implemented using educational interventions such as lectures, group work, discussions, and practical exercises or simulations (Kulju et al. 2024).

Given the heterogeneous background of nurse educators internationally, it is worth examining whether it is possible to identify any uniform groups based on digital competence and whether the groups differ based on educators' background variables. In this way, continuing education could be targeted to the specific groups with the intention of addressing competence needs (Pilcher 2016). Therefore, the aim of this study was to identify and compare the competence profiles of nurse educators in four European countries (Finland, Malta, Slovakia and Spain), with a particular focus on the levels of digital competence within these profiles. With this knowledge, it is possible to understand nurse educators' CPD needs in digital competence in order to implement corresponding continuing education in the international context.

The research questions included the following:

- What digital competence profiles can be identified among nurse educators in four European countries?
- What background variables are associated with the digital competence profiles of nurse educators in four European countries?
- What is the self‐assessed level of digital competence between the competence profiles of nurse educators in four European countries?

## 3 Methods

### 3.1 Study Design and Context

This descriptive comparative cross‐sectional study was carried out as part of a project funded by Erasmus+ (New Nurse Educator ‐project), which aimed to study, develop and harmonise the education of nurse educators in Europe. This study focuses on nurse educators from higher education nursing education organisations from four countries: Finland, Malta, Slovakia and Spain.

There are some dissimilarities between these countries in the educational requirements of educators and in the working environments. For example, a master's degree (European Qualifications Framework [EQF] 7) is sufficient for qualified nurse educators in Finland, Malta and Slovakia, whereas a doctoral degree (EQF 8) is required in Spain. In Finland, Slovakia and Spain, nurse educators' background education is usually in health sciences, and in Malta, it could be in pedagogy, anthropology or sociology. In these four countries, pedagogical studies may be included in the educators' education, as in Finland. However, these studies are not mandatory. In Finland, in contrast to the other three countries, nurse educators are required to have at least 3 years of clinical work experience. In these four countries, clinical teaching is seen as one part of the educator's work, but development projects, research, and teaching theoretical and practical skills in classrooms, digital environments, and skill labs are more common tasks of nurse educators (Campos Silva et al. 2022).

Moreover, the four countries differ in the length of nursing education study programmes—that is, from 3 years (Malta and Slovakia) to 3.5 (Finland) to 4 (Spain). The sum of ECTS is that various countries correspond to the length of the study (180 ECTS in Malta and Slovakia, 210 ECTS in Finland, and 240 ECTS in Spain) (Directive 2013/55/EU 2013). The study methods used in these countries do not differ themselves (including contact lessons, remote and independent studies, e‐learning, simulation, practice, and research and development work). However, the utilisation of educational technology for using these methods that are essential for the theoretical and practical education might differ in the extent and level of nurse educators' competence (Campos Silva et al. 2022).

### 3.2 Data Collection Instrument

Digital competence was examined using the Educators and Educator Candidates' Competence in Digital Pedagogy (OODI) scale. The OODI was chosen because it is based on the widely utilised DigCompEdu framework (Redecker 2017). In addition, it is a newly validated instrument for assessing nurse educators' digital competence in order to identify competence development needs (Pajari et al. 2024). The OODI includes two visual analogue scale (VAS) items related to the assessment of educators' digital competence and interest in educational technology (from 0 = weak to 5 = strong). Moreover, it consists of 20 items, included in three factors and scored on a Likert scale, which reflects digital competence (1 = not at all, 5 = very well). The first factor (implementing appropriate independent and community learning) consisted of eight items, the second (acting safely and responsibly) included seven items, and the third (guiding learning based on the evidence) consisted of five items. The OODI has high reliability with a Cronbach's *α* value of 0.938 and McDonald *ω* value of 0.940 (DeVellis 2017) in the context of Finnish nurse educators in higher education nursing organisations (Pajari et al. 2024).

The OODI and the nurse educators' background variables (age, highest level of education, clinical work experience, pedagogical studies, participation continuing professional education, and work experience as an educator) have been originally formulated in Finnish and translated into English. During this study, these were further translated into Spanish and Slovak languages. Educators conducted a pretest of the translations in both languages, followed by a back translation into English and, subsequently, into Finnish, to ensure semantic and cultural equivalence. The translations were performed by independent translators, and the research team evaluated the consistency of these translations to ensure the instrument's validity and reliability in the new contexts (DeVellis 2017; Sousa and Rojjanasrirat 2011).

### 3.3 Data Collection

The language versions of the OODI instrument with nurse educators' background variables were distributed through an online survey in the REDCap (the Research Electronic Data Capture) software (Vanderbilt University, Nashville, Tennessee, USA). The project coordinator from the (University of Turku) in Finland was responsible for the data collection. Data were collected from May 2021 to February 2022. First, a link, including information about the study and a privacy notice, was sent to each country's project partner. They then forwarded the survey link to the nursing education organisations' contact persons, who emailed the survey to nurse educators (*N* = 1163) in their country's organisation (total *N* = 36, Finland: 9, Spain: 17, Slovakia: 9, Malta: 1). The sample size was determined by power analysis. Three reminders about participating in the study were sent through the contact persons. Data from nurse educators in the four countries formed one data set in the REDCap software.

## 4 Data Analysis

Data were analysed using R Statistical Software version 4.3.0 with clustering package 2.1.4. McDonald ω coefficients were analysed to verify the OODI instrument internal consistency. Means and standard deviations, as well as frequencies and percentages, were examined from the data. Several clustering methods were used, such as *k*‐means and *k*‐medoids clustering by varying variables, which produced cluster alternatives. Eventually, the previously validated three‐sum variable factor structure of the OODI instrument (Pajari et al. 2024) was utilised in the k‐medoids clustering, partitioning around medoids (PAM) cluster analysis. PAM clustering as a method allows the entry of various forms of variables. In the PAM model, one data point case is selected as a medoid, and the data with the least dissimilarities to the medoid are clustered around it. This accurate method classifies data into clusters based on true dissimilarities instead of the mean and distinguishes the important variables for clustering. This statistical clustering method was used to determine whether nurse educators from four countries belonged to identifiable groups based on their self‐assessed digital competence. The aim of clustering was to identify digital competence profile groups with the least within‐group dissimilarity and maximum between‐group dissimilarity (Kaufman and Rousseeuw 1990; Schubert and Rousseeuw 2021). From several analyses, the final two‐cluster result (*k* = 2) was decided based on a meaningful cluster structure and the most appropriate result of average silhouette width (ASW) *s*(*i*) being 0.44 of the total data set (ASW value for cluster 1 = 0.50, and for cluster 2 = 0.38) (Batool and Hennig 2021; Kaufman and Rousseeuw 1990). The PAM analysis included only full responses; therefore, listwise deletion (*n* = 27) was performed for missing values (Kaufman and Rousseeuw 1990).

Then, nurse educators' background variables and the self‐assessed digital competence between competence profiles were analysed using Pearson's Chi‐squared test, Fisher's Exact test, Pearson residuals, Welch Two Sample *t*‐test and Mann–Whitney *U* test. The level of statistical significance was set to *p* < 0.05 (Field 2018), and Cohen's *d*, odds ratio (OR), and Vargha–Delaney A (VDA) effect sizes were examined to assess the significance of differences between groups. The highest level of education, pedagogical studies, and participation in continuing professional education variables were formed as binary dummy variables (University degree [master's or doctoral degree]/yes = 1, Other degree/no = 0). VAS items were changed for reporting to a scale of 1–5. The following digital competence level descriptions were used in reporting the results: 1.00–1.99 low, 2.00–2.99 tolerable, 3.00–3.99 moderate, and 4.00–5.00 high.

The STROBE checklist was adhered to when reporting the results (STROBE 2024).

### 4.1 Ethical Considerations

*The European Code of Conduct for Research Integrity* (All European Academies, 2017) and *the General Data Protection Regulation* (Regulation EU 2016) were followed during this study. Turku University's ethics committee approved the study (Decision: 5/2021, 16th February 2021), and research permissions were obtained from all participating nursing education organisations. In addition, contact persons delivered information on the study and a privacy notice to nurse educators via email, and educators were informed about voluntary participation. Finally, collected data were treated anonymously, and the original authors authorised the use of the OODI instrument (World Medical Association 2013).

## 5 Results

Nurse educators (*n* = 263) from Finland (38.8%), Malta (6.5%), Slovakia (17.1%) and Spain (37.6%) participated in this study, with a corresponding response rate of 22.6% (Table 1). Educators' average age was 48 years. Almost all had completed a nursing degree and nursing qualification. The majority of the educators had completed a postgraduate university degree (doctorate 44.5% or master's degree 46.8%). Of the nurse educators, 73% had completed pedagogical studies (variation 43.4%–99%), and 80.2% of the educators had participated in continuing professional education (variation 61.7%–100%). The educators had an average of 15.7 (SD 9.8) years of nursing experience and an average of 12.5 (SD 9.40) years of teaching experience as a nurse educator.

**TABLE 1:** Nurse educators' sociographic background variables by country.

	Finland	Malta	Slovakia	Spain	Total
Nurse educators, *n* (%)	102 (38.8)	17 (6.5)	45 (17.1)	99 (37.6)	263 (100)
Age in years, mean (SD)	49.4 (9.2)	50.1 (10.8)	49.0 (8.1)	45.6 (9.2)	47.9 (9.3)
Completed degree in nursing, *n* (%)	102 (100.0)	16 (94.1)	45 (100.0)	94 (94.9)	257 (97.7)
Nursing qualification, *n* (%)	102 (100.0)	17 (100.0)	41 (91.1)	89 (89.9)	249 (94.7)
Highest degree, *n* (%)					
PhD	12 (11.8)	10 (58.8)	40 (88.9)	55 (55.6)	117 (44.5)
Master	76 (74.5)	7 (41.2)	5 (11.1)	35 (35.3)	123 (46.8)
Graduate diploma	10 (9.8)	0	0	6 (6.1)	16 (6.1)
Other	4 (3.9)	0	0	3 (3.0)	7 (2.7)
Pedagogical studies completed, *n* (%)	101 (99.0)	9 (52.9)	39 (86.7)	43 (43.4)	192 (73.0)
Participation in continuing professional education, *n* (%)	63 (61.7)	15 (88.2)	34 (75.6)	99 (100.0)	211 (80.2)
Experience in nursing, mean years (SD)	14.1 (7.3)	16.1 (11.7)	12.9 (11.6)	18.5 (10.2)	15.7 (9.8)
Experience in teaching, mean years (SD)	11.52 (9.5)	15.29 (11.6)	19.24 (10.0)	10.06 (7.0)	12.5 (9.40)

Considering the first research question, PAM clustering analysis identified two cluster groups. These identified cluster groups were named Profile A (High) (mean 4.00–5.00) and Profile B (Moderate) (mean 3.00–3.99), based on the nurse educators' self‐assessed digital competence level (OODI total). Profile A described the high level of digital competence and included 122 (46.4%) of the nurse educators from the four countries. Competence profile B, where 141 (53.6%) of the educators were included, described the moderate digital competence (Table 2).

**TABLE 2:** The competence profiles of digital competence of nurse educators in four European countries using PAM clustering.

	Profile A (high)	Profile B (moderate)	*p*
Educators (*n*, %)	122 (46.4)	141 (53.6)	
Country (*n*, %)			
Finland	47 (46.1)	55 (53.9)	0.316^a^
Malta	6 (35.3)	11 (64.7)	
Spain	43 (43.4)	56 (56.6)	
Slovakia	26 (57.8)	19 (42.2)	
Age in years (*n*, %)			
≤ 40	23 (46.9)	26 (53.1)	0.586^a^
> 40–50	46 (47.9)	50 (52.1)	
> 50–60	35 (42.2)	48 (57.8)	
> 60	17 (56.7)	13 (43.3)	
Mean (SD)	47.9 (9.25)	47.9 (9.31)	1.000^b^
Min–max	29–67	24–64	
Nursing education (*n*, %)			
Yes	119 (46.3)	138 (53.7)	1.000^c^
No	3 (50.0)	3 (50.0)	
Nursing qualification (*n*, %)			
Yes	116 (46.6)	133 (53.4)	1.000^c^
No	5 (45.5)	6 (54.5)	
Highest degree (*n*, %)			
Doctoral	61 (52.1)	56 (47.9)	n.a.^d^
Masters	55 (44.7)	68 (55.3)	
Graduate degree	6 (37.5)	10 (62.5)	
Other	0 (0.0)	7 (100.0)	
Clinical nursing experience in years (*n*, %)			
≤ 5	19 (50.0)	19 (50.00)	0.232^a^
> 5–10	38 (55.1)	31 (44.9)	
> 10–15	14 (40.0)	21 (60.0)	
> 15–20	15 (34.1)	29 (65.9)	
> 20	33 (47.8)	36 (52.2)	
Mean	14.9 (10.0)	16.3 (9.55)	0.144^e^
Min–max	0–40	0–42	
Pedagogical studies completed (*n*, %)			
Yes	97 (50.5)	95 (49.5)	**0.037** ^c^
No	25 (35.7)	45 (64.3)	
Continuing education courses after graduation (*n*, %)			
Yes	103 (48.8)	108 (51.2)	0.257^c^
No	18 (39.1)	28 (60.9)	
Teaching experience in years (*n*, %)			
≤ 5	29 (38.7)	46 (61.3)	0.287^a^
> 5–10	25 (48.1)	27 (51.9)	
> 10–15	22 (40.7)	32 (59.3)	
> 15–20	16 (57.1)	12 (42.9)	
> 20	28 (53.9)	24 (46.1)	
Mean (SD)	13.9 (9.88)	11.3 (8.84)	**0.035** ^e^
Min–max	0–45	0–39	

*Note:* Bold values indicate *p* < 0.05.

^a^ Pearson's Chi‐squared test.

^b^ Welch Two Sample *t*‐test.

^c^ Fisher's Exact test.

^d^ Pearson residuals.

^e^ Mann–Whitney *U* test.

For the second research question, the sociographic background variables of nurse educators were associated with digital competence profiles. Of the educators included in the high digital competence profile, 79.5% had completed pedagogical studies, whereas 31.9% had not completed pedagogical studies in the moderate competence profile (*p* = 0.037, OR 0.545). Also, educators in the high competence profile had more years of teaching experience (mean 13.9 [SD 9.88]) than those in the moderate profile (mean 11.3 [SD 8.84]) (*p* = 0.035, VDA 0.566) (Table 2).

Finally, to address the last research question, there was a significant difference among the two digital competence profiles of the nurse educators from the four countries. Educators in the high competence profile perceived the VAS evaluation of their own digital competence to be higher than those in the moderate competence profile (*p* < 0.001, Cohen's *d* 1.023). In addition, they were more interested in educational technology than educators on a moderate profile (*p* < 0.001, VDA 0.742). With the OODI instrument, educators in the high competence profile self‐assessed their digital competence as being at a higher level in all three OODI factors (*p* < 0.001) and in the OODI total assessment (*p* < 0.001), as compared to those in the moderate competence profile (Table 3).

**TABLE 3:** Self‐assessed digital competence level of nurse educators in four countries between competence profiles.

Digital competence (mean, SD)	McDonald *ω*	Total	Profile A (high)	Profile B (moderate)	*p*	Standardised effect size
Self‐assessment of digital competence^a^		3.83 (0.63)	4.13 (0.45)	3.56 (0.64)	**< 0.001** ^b^	1.023^c^
Self‐assessment of interest in educational technology^a^		4.23 (0.63)	4.39 (0.51)	4.09 (0.69)	**< 0.001** ^d^	0.742^e^
OODI implementing appropriate independent and community learning^f^	0.884	3.78 (0.62)	4.25 (0.39)	3.38 (0.48)	**< 0.001** ^d^	0.937^e^
OODI acting safely and responsibly^f^	0.887	3.33 (0.79)	3.92 (0.51)	2.81 (0.60)	**< 0.001** ^b^	1.986^c^
OODI guiding learning based on the evidence^f^	0.895	3.43 (0.75)	4.05 (0.46)	2.90 (0.50)	**< 0.001** ^d^	0.975^e^
OODI total^f^	0.955	3.51 (0.64)	4.07 (0.36)	3.03 (0.39)	**< 0.001** ^b^	2.768^c^

^a^ Scale of VAS items in reporting 1–5.

^b^ Welch Two Sample *t*‐test.

^c^ Cohen's *d* effect size.

^d^ Mann–Whitney *U* test.

^e^ Vargha–Delaney A effect size.

^f^ Likert scale of 1–5.

In both clusters, the highest competence level was in utilising technology to support their own CPD, as 72.3% of educators self‐assessed using technology well or very well for this. The weakest competence level for the nurse educators was in guiding their students to protect their own produced digital material (cluster A mean 3.6 [SD 0.9]; cluster B mean 2.4 [SD 0.9]) (Tables 1 and S1).

## 6 Discussion

This study has described the digital competence profiles of nurse educators in four European countries, the background variables associated with profiles, and the level of digital competence. This knowledge can be used to target continuing education so that nurse educators may enhance their digital competence as part of CPD.

With PAM clustering, it was possible to identify two distinct groups of nurse educators based on self‐assessed digital competence. These groups have been described as having profiles of the high digital competence level and the moderate digital competence level. This is an interesting finding, considering nationally and internationally heterogeneous educators' education regulations (Satoh et al. 2020; Leighton et al. 2022; Wells‐Beede et al. 2023) and the diversity of competence descriptions (Campos Silva et al. 2022; Fitzgerald et al. 2020). Even though nurse educators in Finland, Malta, Slovakia and Spain cannot be treated as one similar group due to this heterogeneity, it seems they can be seen as two distinct groups based on their self‐assessed digital competence. Identifying these competence profiles was important in making digital competence and the associated variables more visible and understandable and in further implementing common continuing education (Drude et al. 2019; Jobst et al. 2022; Pilcher 2016) and educators' international cooperation (Directive 2013/55/EU 2013; Filej et al. 2024; WHO 2021).

When comparing educators' background variables, competence profiles differed significantly regarding the completed pedagogical studies and teaching experience. This emphasis on pedagogical studies in the high digital competence profile is very understandable because these studies are deemed important for educators' competence (Breytenbach et al. 2017; de Gagne 2022; Oermann 2022; Zlatanovic et al. 2017). Through pedagogical studies, nurse educators have the evidence‐based competence to combine theoretical knowledge with hands‐on teaching. With pedagogical studies, educators have acquired the competence to plan, implement, and assess teaching, learning, and curriculum in various educational environments. For example, through pedagogical studies, nurse educators have the competence to consider the usefulness and accessibility of digital materials and technology for student groups and individual students (Ryhtä et al. 2021; Vähäkangas et al. 2022; Zhao et al. 2021). In this way, they have versatile insights on how to integrate technology into today's nursing education (de Gagne 2022), which may appear as an association with the high competence profile. On the other hand, with pedagogical studies, educators are perhaps more aware of the educator's competence requirements as compared to WHO's (2016) more country‐specific ones (e.g., Mikkonen et al. 2021). Thus, they have been able to identify and reflect on their own competence (Koskimäki et al. 2022). This reflection can be seen as one part of CPD (Drude et al. 2019), and this may also explain the connection to a high profile of digital competence.

In addition to pedagogical studies, an interesting result was the association of greater teaching experience in the high digital competence profile group. This can be considered an expected result because, through experience, educators have had the opportunity to adapt several technology‐based teaching, learning and assessment methods in varying contexts (Jobst et al. 2022; Lee and Bello 2023; Vardaman et al. 2024), such as digital learning environments in remote learning (Jämsä et al. 2024). They have also accumulated expertise in assessing students' needs and the appropriate methods for them. In this way, with prolonged educational experience, educators have had the opportunity in CPD to continuously acquire a more comprehensive competence to evaluate and implement meaningful technology for various educational situations (Breytenbach et al. 2017; de Gagne 2022; Oermann 2022; Zlatanovic et al. 2017). In addition, with longer experience, educators may have had the opportunities in cooperation with peers in several communities to develop and share experiences of the implementation of educational technology (Filej et al. 2024; Gazza 2019; Lee and Bello 2023). Because novice nurse educators with less teaching experience are being pressured to learn multidimensional educators' competencies, such as pedagogical and clinical nursing knowledge in addition to digital competence (Fitzgerald et al. 2020; Koskimäki et al. 2022; NLN 2022; Zlatanovic et al. 2017), it would be worthwhile to share experienced educators' views, for example, through mentoring less experienced colleagues (Leighton et al. 2022; Vardaman et al. 2024).

Educators in the high competence profile assessed digital competence significantly higher in all areas than those in the moderate competence profile, and they are also more interested in utilising educational technology. There are fewer similar earlier comparisons of educators' digital competence, without identified differences between the educator groups (Boté‐Vericad et al. 2023; Santos et al. 2022; Zhao et al. 2021). Still, in a somewhat similar way, some studies (Jarva et al. 2024; Kaarlela et al. 2022; Kaihlanen et al. 2023) have identified healthcare professionals' competence profiles, such as those of nurses and clinical educators whose ability to utilise technology was related to high competence profile. This reinforces the knowledge that professionals working in healthcare have various competence strengths and interests that can be identified (Jarva et al. 2024; Kaarlela et al. 2022; Kaihlanen et al. 2023). This knowledge can be used, for example, by modifying work tasks to match competence. This could mean that technology‐oriented educators would take responsibility for technology development and become mentors to promote the utilisation of technology in the work community (Leighton et al. 2022). On the other hand, educators with a high level of digital competence could participate either in more advanced continuing education regarding digital competence or in some other CPD activities that better correspond to their development needs (Drude et al. 2019; Pilcher 2016).

Nurse educators included in the moderate competence profile could be targeted with continuing education to enhance their digital competence. Although all educators would certainly benefit from the education (Vardaman et al. 2024), when the resources of the organisation and the educators are limited, it may be most effective to prioritise the continuing education for those who will benefit the most (Brewer et al. 2024). In this way, continuing education can be developed more deeply to deal with those areas of competence where competence is at a lower level. For example, educators with the moderate digital competence profile could set their CPD learning objectives in the safe and responsible use of technology to promote knowledge of copyright laws (Redecker 2017). This competence is especially important to recognise and highlight because an increasing amount of digital materials is currently being used, shared and edited in a variety of environments in nursing education and the clinical practice of healthcare. The use of these materials, such as videos or registry data, involves significant copyright and security considerations that educators should recognise. Concurrently, they require the competence to teach these copyright issues to students (OECD 2023; WHO 2021).

Further, no other nurse educators' background variables were significantly related to the competence profiles. It seems that educators who have completed continuing education have a slightly greater emphasis on a higher digital competence profile. This could be logical if education has, for example, dealt with areas of digital competence (Kulju et al. 2024), but this is not often the case. Because continuing education is fragmented, there would be an opportunity in the future to harmonise and offer continuing education according to digital competence needs (Smith et al. 2023; Tischendorf et al. 2024).

Also, most Slovak educators (57.8%) seem to belong to the profile of high digital competence, whereas a larger proportion of Finnish (53.9%), Maltese (64.7%) and Spanish (56.6%) educators seem to be in the profile of moderate competence. It will be important to learn more about this variation in digital competence between countries. It may be that educators in certain countries require more awareness of the possibilities of educational technology and therefore self‐assessed their digital competence either too positively or critically (de Gagne 2022; Filej et al. 2024; Lee and Bello 2023). Moreover, Slovak educators in this study often had a doctoral degree. Maybe educators' varying education regulations or curriculum contents could explain differences in competence between countries (Campos Silva et al. 2022; Fitzgerald et al. 2020).

Furthermore, without significant difference, nurse educators in a higher digital competence profile had more doctoral degree level backgrounds, who at the same time had less clinical experience. This may be because educators who have continued their studies to the doctoral level may have had a shorter period to gain clinical experience. Higher digital competence level can be explained by the fact that, during their doctoral studies, they may have also studied evidence‐based teaching and utilised educational technology (Breytenbach et al. 2017; Oermann 2022; Vardaman et al. 2024). In the moderate competence profile, educators had more clinical competence. Due to this, they may have had less possibilities to familiarise themselves with the use of technology in the educational context, explaining the identified digital competence level (Jobst et al. 2022; Vardaman et al. 2024). The absence of nurse educators' nursing education and qualification was slightly emphasised in the higher competence profile. This can be explained by the fact that it is possible to work in some countries as a nurse educator with a degree in another field (Campos Silva et al. 2022). For example, in Malta, a postgraduate degree may be in pedagogy or anthropology (Campos Silva et al. 2022), which perhaps explains the educators' more comprehensive pedagogical competence in utilising technology in education (Vähäkangas et al. 2022). In the future, it would be interesting to learn about the competence levels of nurse educators' other competence areas, such as nursing competence (Fitzgerald et al. 2020; Mikkonen et al. 2021; NLN 2022; WHO 2016), in these identified digital competence profiles.

Finally, the nurse educators' age in this study did not significantly differ in the competence profiles. This can be explained by the fact that, despite their age, educators have had to utilise educational technology during the digitalisation of healthcare education, partly attributed to the COVID‐19 pandemic (Filej et al. 2024; Jämsä et al. 2024; OECD 2023). Or, as has been observed from healthcare professionals, the more extensive use of technology in work and leisure time, as well as the organisation's support (Jarva et al. 2024) and sufficient orientation (Kaihlanen et al. 2023), could have positively affected nurse educators' competence and attitude in using and adopting technology. As Lee and Bello (2023) noted, individual variables rarely define competence holistically. The importance of these different background variables for nurse educators' digital competence should be examined in the future to understand and develop competence in CPD activities more comprehensively.

Based on the results of this study and confirmed by previous evidence, some recommendations for nurse educators' CPD can be suggested to enhance educators' digital competence. Continuing education as part of CPD could be targeted to include safe and responsible technology use, such as enhancing educators' competence to guide students in protecting produced digital materials (Redecker 2017). As identified in the results, most educators assessed that they could use technology to support their own CPD. Considering this, continuing education could be implemented with a multimethod approach, including individual, needs‐based guidance. Competence should be enhanced through practical education with interaction opportunities implementing technology (Kulju et al. 2024). The implementation of education and development of competence should be assessed using several methods, such as objective assessment in addition to self‐assessment, while also considering resources (Marceau et al. 2024).

In addition, for educators lacking in pedagogical studies, CPD could be focused on pedagogical foundations to implement evidence‐based education. This would support the enhancement of digital competence in utilising effective educational approaches with technology (Breytenbach et al. 2017; Männistö et al. 2020; Sormunen et al. 2022). For those with less teaching experience, peer mentoring could be developed to support the utilisation of technology in teaching, learning, and assessment (Leighton et al. 2022; Smith et al. 2023). Because nurse educators expect continuing education to meet the competence needs revealed by ongoing trends (Koskimäki et al. 2022), such as artificial intelligence (Xia et al. 2024), these could be included in the nurse educators' CPD learning opportunities (Drude et al. 2019; Krumsvik 2022; Vähäkangas et al. 2022).

## 7 Limitations and Strengths

The study's results are based on the educators' self‐assessment, so it may be possible that the assessment's focus has been influenced by the educators' self‐reporting of their digital competence. For example, it may be that the educators assessed their previously accustomed pedagogical and teaching approaches instead of the versatile and innovative use of educational technology (Field 2018), even though technology was included in the items of the OODI instrument (DeVellis 2017). There is a need to examine nurse educators' digital competence with several methods, such as observing educators' actual use of technology.

The strength of the study was that the basis of the OODI instrument (Pajari et al. 2024) and the view of digital competence in this study were aligned on the widely adopted DigCompEdu framework (Redecker 2017; Zhao et al. 2021). In addition, data were collected in cooperation with experienced researchers, consisting of nurse educators, from four European countries. The respondents represented nurse educators from several organisations located in the four target countries of the study. McDonald *ω* coefficient showed good reliability for the OODI instrument (DeVellis 2017). The data were assessed as decent for the methods used (Batool and Hennig 2021; Field 2018; Kaufman and Rousseeuw 1990; Schubert and Rousseeuw 2021).

Moreover, the heterogeneity in national contexts and education regulations for nurse educators in the countries studied may limit generalisation of the results. Each country has different educational requirements and work expectations, which may influence the interpretation and utilisation of the identified digital competence profiles. Nevertheless, this international approach, covering four European countries, is a strength, allowing a comparative understanding of the digital competences of nurse educators in different educational and cultural contexts. Considering the response rate (22.6%, *n* = 263), the results can be used as part of the development of internationally common CPD activities for nurse educators.

## 8 Conclusion

Despite the heterogeneity in the education and competence of nurse educators across four European countries, this study identified two distinct digital competence profiles: high and moderate. These competence profiles clarify knowledge about the background variables related to digital competence, allowing CPD activities to be targeted to meet educators' competence needs. Continuing education programmes for nurse educators could benefit from focusing on the safe and responsible use of technology, such as the protection of sensitive data, which is critical for the ethical management of digital materials in educational and practical settings. Continuing education could be implemented in practice through a more technological focus, as nurse educators assessed themselves to be well prepared to use technology in support of their own CPD. Moreover, nurse educators' CPD activities could include experienced educators mentoring peers with less teaching experience. To further foster more advanced digital competence, it is advisable for CPD programmes to include pedagogical approaches that teach educators to integrate educational technology into evidence‐based teaching, learning, and assessment methods. Such programmes could focus on teaching strategies that utilise technology to enrich the learning experience, personalise instruction, and enhance student assessment. This enables educators to develop a more flexible, student‐centred pedagogical approach that is better suited to individual student needs.

## Author Contributions

Made substantial contributions to conception and design, or acquisition of data, or analysis and interpretation of data: J.P., M.S., L.S., I.E., M.P., L.M.‐D., M.C., A.S., E.H.‐S. and T.S. Involved in drafting the manuscript or revising it critically for important intellectual content: J.P., M.S., L.S., I.E., M.P., L.M.‐D., M.C., A.S., E.H.‐S. and T.S. Given final approval of the version to be published. Each author should have participated sufficiently in the work to take public responsibility for appropriate portions of the content: J.P., M.S., L.S., I.E., M.P., L.M.‐D., M.C., A.S., E.H.‐S. and T.S. Agreed to be accountable for all aspects of the work in ensuring that questions related to the accuracy or integrity of any part of the work are appropriately investigated and resolved: J.P., M.S., L.S., I.E., M.P., L.M.‐D., M.C., A.S., E.H.‐S. and T.S.

## Disclosure

The authors have checked to make sure that our submission conforms as applicable to the Journal's statistical guidelines. Miko Pasanen, mikos.pasanen@gmail.com, is the author team's statistician. The authors affirm that the methods used in the data analyses are suitably applied to data within the study design and context, and the statistical findings have been implemented and interpreted correctly. The authors agree to take responsibility for ensuring that the choice of statistical approach is appropriate and is conducted and interpreted correctly as a condition to submit to the Journal.

## Ethics Statement

The study was approved by the Ethics Committee of University of Turku (Decision: 5/2021, 16th February 2021).

## Consent

Electronic informed consent was obtained from all nurse educators before participating in the study.

## Conflicts of Interest

The authors declare no conflicts of interest.

## Supporting information


Table S1.

Data S1.

## Data Availability

The data of this study, without personal identifiers, will be available in the Finnish Social Science Data Archive (FSD) after the results of the New Nurse Educator project have been published.
