# Supplementary material for: Nurse Educators' Background, Education, and Experience in Digital Competence Profiles: A Descriptive Comparative Cross‐Sectional Study in Four Countries
Source: J Adv Nurs. 2025 Jul 15;82(4):3172–83. doi: 10.1111/jan.70077 (PMC12994616; doi:10.1111/jan.70077)
Supplement: Supplementary file 1 — Table S1. [file JAN-82-3172-s001.pdf]

Supplementary Material 1, Table 1. Frequencies and percentages of nurse educators' responses to the items of the OODI instrument in competence profiles.

	1<br>f (%)	2<br>f (%)	3<br>f (%)	4<br>f (%)	5<br>f (%)	Total mean (SD)
**Factor 1. Implementing appropriate independent and community learning**						
Using technology to guide learners						
Cluster A	0 (0)	0 (0)	5 (7.5)	74 (56.1)	43 (87.8)	4.3 (0.5)
Cluster B	2 (100)	13 (100)	62 (92.5)	58 (43.9)	6 (12.2)	3.4 (0.8)
Total	2 (0.8)	13 (4.9)	67 (25.5)	132 (50.2)	49 (18.6)	3.8 (0.8)
Using digital resources to support my own continuing professional development.						
Cluster A	0 (0)	3 (25)	4 (6.9)	67 (50.8)	48 (82.8)	4.3 (0.7)
Cluster B	3 (100)	9 (75)	54 (93.1)	65 (49.2)	10 (17.2)	3.5 (0.8)
Total	3 (1.1)	12 (4.5)	58 (22.1)	132 (50.2)	58 (22.1)	3.9 (0.8)
When choosing digital resources, considering their suitability for teaching and learning						
Cluster A	0 (0)	0 (0)	11 (14.3)	82 (57.3)	29 (87.9)	4.1 (0.6)
Cluster B	0 (0)	10 (100)	66 (85.7)	61 (42.7)	4 (12.1)	3.4 (0.7)
Total	0 (0)	10 (3.8)	77 (29.3)	143 (54.4)	33 (12.5)	3.8 (0.7)
Using technology in teaching to enable collaborative learning.						
Cluster A	0 (0)	0 (0)	6 (6.8)	91 (71.7)	25 (89.3)	4.2 (0.5)
Cluster B	2 (100)	18 (100)	82 (93.2)	36 (28.3)	3 (10.7)	3.1 (0.7)
Total	2 (0.8)	18 (6.8)	88 (33.5)	127 (48.3)	28 (11.6)	3.6 (0.8)
Supporting learners' self-direction through technology.						
Cluster A	0 (0)	2 (5.7)	12 (13.2)	83 (74.8)	25 (100)	4.1 (0.6)
Cluster B	1 (100)	33 (94.3)	79 (86.8)	28 (25.2)	0 (0)	3.0 (0.7)
Total	1 (0.4)	35 (13.3)	91 (34.6)	111 (42.2)	25 (9.5)	3.5 (0.9)
Evaluating of own digital competence.						
Cluster A	0 (0)	2 (11.8)	7 (12.7)	73 (53.3)	40 (78.4)	4.2 (0.6)
Cluster B	3 (100)	15 (88.2)	48 (87.3)	64 (46.7)	11 (21.6)	3.5 (0.9)
Total	3 (1.1)	17 (6.5)	55 (20.9)	137 (52.1)	51 (19.4)	3.8 (0.9)
Producing digital learning materials, considering their suitability for teaching and learning.						
Cluster A	0 (0)	1 (3.7)	13 (16.7)	75 (63)	33 (86.8)	4.1 (0.6)
Cluster B	1 (100)	26 (96.3)	65 (83.3)	44 (37)	5 (13.2)	3.2 (0.8)
Total	1 (0.4)	27 (10.3)	78 (29.7)	119 (45.3)	38 (14.4)	3.6 (0.9)
**Factor 2. Acting safely and responsibly**						
Guiding learners to protect the material they produce in accordance with copyright law.						
Cluster A	5 (17.2)	6 (10.2)	38 (44.2)	54 (79.4)	19 (90.5)	3.6 (0.9)
Cluster B	24 (82.8)	53 (89.8)	48 (55.8)	14 (20.6)	2 (9.5)	2.4 (0.9)
Total	29 (11)	59 (22.4)	86 (32.7)	68 (25.9)	21 (8)	3.0 (1.1)
Protecting the privacy of sensitive digital material used in teaching, such as videos recorded in learning situations and used to analyze student activity.						
Cluster A	1 (11.1)	3 (7.7)	15 (24.6)	62 (61.4)	41 (77.4)	4.1 (0.8)
Cluster B	8 (88.9)	36 (92.3)	46 (75.4)	39 (38.6)	12 (22.6)	3.1 (1.0)
Total	9 (3.4)	39 (14.8)	61 (23.2)	101 (38.4)	53 (20.2)	3.6 (1.1)
Using digitally licensed digital materials in the right way.						
Cluster A	1 (9.1)	2 (6.5)	16 (22.9)	65 (61.3)	38 (84.4)	4.1 (0.8)
Cluster B	10 (90.1)	29 (93.5)	54 (77.1)	41 (38.7)	7 (15.6)	3.0 (1.0)
Total	11 (4.2)	31 (11.8)	70 (26.6)	106 (40.3)	45 (17.1)	3.5 (1.0)
Supporting learners to produce responsible digital content.						
Cluster A	1 (10)	6 (15)	27 (25.5)	66 (77.7)	22 (100)	3.8 (0.8)
Cluster B	9 (90)	34 (85)	79 (74.5)	19 (22.3)	0 (0)	2.8 (0.8)
Total	10 (3.8)	40 (15.2)	106 (40.3)	85 (32.3)	22 (8.4)	3.3 (0.9)
Guiding learners to the critical use of digital media.						
Cluster A	0 (0)	1 (3.1)	13 (17.8)	78 (65)	30 (90.9)	4.1 (0.6)

Cluster B	5 (100)	31 (96.9)	60 (82.2)	42 (35)	3 (9.1)	3.0 (0.9)
Total	5 (1.9)	32 (12.2)	73 (27.8)	120 (45.6)	33 (12.5)	3.5 (0.9)
Supporting learners' ability to solve problems with technology.						
Cluster A	0 (0)	4 (8.9)	35 (30.7)	70 (89.7)	13 (100)	3.8 (0.7)
Cluster B	13 (100)	41 (91.1)	79 (69.3)	8 (10.3)	0 (0)	2.6 (0.7)
Total	13 (4.9)	45 (17.1)	114 (43.4)	78 (29.7)	13 (4.9)	3.1 (0.9)
Guiding the development of learners' information literacy in a digital learning environment.						
Cluster A	0 (0)	2 (4.5)	28 (27.7)	76 (81.7)	16 (94.1)	3.9 (0.6)
Cluster B	8 (100)	42 (95.5)	73 (72.3)	17 (18.3)	1 (5.9)	2.7 (0.8)
Total	8 (3)	44 (16.7)	101 (38.4)	93 (35.4)	17 (6.5)	3.3 (0.9)
**Factor 3. Guiding learning based on the evidence**						
Using technology to give feedback to learners.						
Cluster A	0 (0)	1 (3.6)	10 (12.4)	78 (69)	33 (91.7)	4.2 (0.6)
Cluster B	5 (100)	27 (96.4)	71 (87.6)	35 (31)	3 (8.3)	3.0 (0.8)
Total	5 (1.9)	28 (10.6)	81 (30.8)	113 (43)	36 (13.7)	3.6 (0.9)
Using technology to assess learning according to different assessment methods.						
Cluster A	0 (0)	1 (3.3)	19 (18.6)	74 (75.5)	28 (93.3)	4.1 (0.6)
Cluster B	3 (100)	29 (96.7)	83 (81.4)	24 (24.5)	2 (6.7)	3.0 (0.7)
Total	3 (1.1)	30 (11.4)	102 (38.8)	98 (37.3)	30 (11.4)	3.5 (0.9)
Using learning analytics to assess the progress of learning.						
Cluster A	1 (9.1)	2 (4.3)	33 (30)	61 (85.9)	25 (100)	3.9 (0.8)
Cluster B	10 (90.9)	44 (95.7)	77 (70)	10 (14.1)	0 (0)	2.6 (0.7)
Total	11 (4.2)	46 (17.5)	110 (41.8)	71 (27)	25 (9.5)	3.2 (1.0)
Using technology in individualized teaching.						
Cluster A	0 (0)	1 (3.5)	11 (11.3)	86 (78.2)	24 (100)	4.1 (0.6)
Cluster B	3 (100)	28 (96.5)	86 (88.7)	24 (21.8)	0 (0)	2.9 (0.7)
Total	3 (1.1)	29 (11)	97 (36.9)	110 (41.9)	24 (9.1)	3.5 (0.9)
Motivating learners in a digital environment.						
Cluster A	0 (0)	0 (0)	15 (16)	84 (74.3)	23 (95.8)	4.1 (0.6)
Cluster B	2 (100)	30 (100)	79 (84)	29 (25.7)	1 (4.2)	3.0 (0.7)
Total	2 (0.8)	30 (11.4)	94 (35.7)	113 (43)	24 (9.1)	3.5 (0.8)

Scale: 1 = Not at all, 2 = Poorly, 3 = Neither poorly nor well, 4 = Well, 5 = Very well
